# Optimization of artificial intelligence models for prediction of new-onset cardiovascular disease in patients with arterial hypertension

**DOI:** 10.1371/journal.pdig.0001441

**Published:** 2026-05-21

**Authors:** Enrique Rodilla, Olast Arrizibita-Iriarte, Blanca Miranda-Serrano, Luis-Miguel Ruilope-Urioste, Germán Sedano-Gil, Alberto Ortiz, José-Antonio Costa-Muñoz, José Chordá-Ribelles, Maialen Zabalza-Zudaire, Onintza Sayar-Beristain

**Affiliations:** 1 Department of Internal Medicine, Hypertension and Vascular Risk Unit, Hospital de Sagunto, Foundation for the Promotion of Health and Biomedical Research in the Valencian Region (FISABIO), Valencia, Spain; 2 Departamento de Medicina y Cirugía, Facultad de Ciencias de la Salud, Universidad Cardenal Herrera-CEU, CEU Universities, Alfara del Patriarca, Valencia, Spain; 3 NNBi 2020 S.L, Esquiroz (Galar), Spain; 4 Fundación Renal Íñigo Álvarez de Toledo (FRIAT), Madrid, Spain; 5 Servicio de Nefrología, Hospital 12 de Octubre, Madrid, Spain; 6 Department of Nephrology and Hypertension and RICORS2040, IIS-Fundacion Jimenez Diaz UAM, Madrid, Spain; 7 Department of Preventive Medicine and Public Health, School of Medicine, University of Valencia, Valencia, Spain; 8 Department of Internal Medicine, Consorci Hospital General Universitari de València, Valencia, Spain; Southeast University, CHINA

## Abstract

Advanced preventive strategies are needed to decrease the burden of cardiovascular disease (CVD). We aimed to develop a predictive tool to identify individuals at higher CVD risk and facilitate proactive interventions to improve clinical outcomes. This single-center retrospective study enrolled consecutive hypertensive subjects free of CVD at baseline and followed them up for a mean of 8.3 years. The primary outcome was new-onset CVD (ischemic heart disease, stroke or hospitalization due to heart failure). The 155-variable dataset was enriched by creating trend variables using statistical measures, Principal Component Analysis (PCA) and Latent Class Analysis (LCA). Then, an artificial intelligence (AI) XGBoost prediction algorithm was trained on 70% of the dataset and validated on the remaining 30%. XGBoost-based risk stratification was compared with risk stratification according to SCORE2. The 3,588 consecutive patients enrolled had a mean age of 54.2 ± 14 years, 53% were women. The incidence rate of new-onset CVD was 1.93 (95% CI: 1.78-2.09) per 100 patient-years. The XGBoost model incorporated 30 variables and achieved 86% ROC AUC, 81% sensitivity, and 78% specificity for predicting CVD. The number of antihypertensive drugs had the strongest predictive power within the model. SCORE2 classified at baseline only 32% of participants with a CV event in the follow-up as high or very-high risk, whereas the XGBoost model correctly identified 81% of them. AI-based modeling outperformed SCORE2 in predicting new-onset CVD in patients with hypertension, identifying the number of antihypertensive drugs as a key predictor and supporting the role of AI risk stratification in clinical practice to implement precision medicine.

## Author summary

Cardiovascular disease (CVD) remains a leading cause of illness and death, highlighting the need for better ways to identify people at high risk before problems occur. In this study, we developed and tested an artificial intelligence (AI) tool to improve prediction of new-onset CVD in people with high blood pressure but no prior cardiovascular disease.

We followed 3,588 hypertensive patients for an average of 8 years. Using 155 clinical variables—including laboratory values, medical history, and treatment information—we trained an AI model (XGBoost) to recognize patterns associated with future heart disease, stroke, or heart failure. The model was tested on a separate group of patients to ensure reliability.

The AI tool predicted cardiovascular events with high accuracy (86% overall performance). Notably, the number of blood pressure medications a patient required was one of the strongest predictors of future risk. Compared with the commonly used SCORE2 risk calculator, which identified only one third of patients who later developed CVD as high risk, the AI model correctly identified 81%.

These findings suggest that AI-based risk prediction may help clinicians detect high-risk patients earlier and tailor preventive care more effectively.

## Introduction

Cardiovascular disease (CVD) continues to be the leading cause of death globally, and poses a major public health challenge. According to the World Health Organization (WHO), CVD is responsible for 31% of all deaths worldwide, highlighting the importance of effective strategies for its prevention [1]. Primary prevention, defined as the implementation of measures that prevent the appearance of these diseases in healthy individuals, is the key to reducing the burden of these pathologies. Artificial intelligence (AI) has emerged as a promising tool in this area, owing to its ability to process large volumes of data. Using machine learning algorithms and models, AI can identify patterns of association and predict cardiovascular events, enabling an early intervention for primary prevention [2].

One example is the use of AI systems in precision medicine, in which genetic, environmental, and lifestyle data are analyzed to design personalized intervention plans. Through the analysis of extensive and complex databases, AI can identify early CV risk patterns that have not been previously defined to more effectively implement CVD primary prevention strategies, based on traditional CVRF. The latter includes arterial hypertension (HTN), dyslipidemia, smoking, diabetes and obesity, with hypertension being the most determining variable among the known factors [3]. Early identification of individuals at high risk of developing CVD allows for the implementation of preventive interventions, such as lifestyle changes and pharmacological treatment [4].

AI-based systems can process large amounts of health data, such as electronic health records, laboratory tests, and physical activity records, to identify patterns that might go unnoticed by healthcare professionals. AI models can predict CV diseases based on risk factors by analyzing clinical and biometric data with accuracy comparable to that of experienced cardiologists [5]. In recent years, several studies have demonstrated the usefulness of AI in specific aspects of CVD, such as diabetic retinopathy [6], primary prevention of ischemic heart disease [7] or vascular age and mortality risk [8]. Focusing specifically on the hypertensive population, three studies have recently been published that apply AI/ML models to hypertensive cohorts and focus on prediction of CV events (hospitalization, mortality, or CVD onset), showing that AI approaches outperformed traditional statistical models (logistic regression or baseline algorithms), supporting claims of improved predictive performance [9–11].

This article explores how the proposed AI-based prediction model directly supports a precision-medicine approach in patients with arterial hypertension, by enabling individualized cardiovascular risk stratification beyond conventional risk scores. In particular, we analyze how the model’s ability to integrate multidimensional clinical data may inform personalized prevention and monitoring strategies for new-onset cardiovascular disease. Our objective was to develop a prediction model for early identification of hypertensive patients at high risk for new-onset CV disease, defined as acute myocardial infarction, angina, stroke (ischemic, hemorrhagic and transient ischemic attacks, TIA), and admission for heart failure, in subjects previously free of CVD. The translational relevance of personalized monitoring strategies and preventive interventions will be discussed and compared to standard, available prediction tables, as processing clinical data, biomarkers and established risk factors in AI-models should facilitate integrated clinical workflows.

## Materials and methods

### Study population

This retrospective study included all patients who were treated consecutively in the Arterial Hypertension and Vascular Risk Unit of a regional hospital from March 13, 1991 to March 23, 2023, who met the following inclusion criteria: age between 18 and 75 years, clinical systolic blood pressure (BP) ≥ 140 mmHg and/or clinical diastolic BP ≥ 90 mmHg during one month of follow-up, that is, elevated values in at least two office visits at 1– 4-week intervals using the average of the last two out of three readings per visit, or be receiving antihypertensive treatment at baseline; minimum time of evolution and follow-up of one year in the study; and availability of at least two visits with the clinical and biochemical data necessary for the assessment of cardiovascular risk (CVR). In the vast majority of cases patients were referred by the Family physicians to confirm the diagnosis of hypertension and to manage follow-up. The index (baseline) time was defined as the first eligible visit to the Hypertension and Vascular Risk Unit that met the inclusion criteria. Follow-up began at baseline and continued until the last measurement prior to the cardiac event (never post-event information), death, or administrative termination of follow-up (March 23, 2023), whichever occurred first. The BP measurement process, clinical management, and complementary tests have been described previously [12]. The glomerular filtration rate (eGFR) was estimated from serum creatinine using the MDRD equation. We excluded patients who presented with secondary hypertension, neoplastic or systemic disease, or hepatic or renal failure (glomerular filtration rate <30 ml/min/1.73 m² and/or clinical proteinuria > 300 mg/24h), heart failure (New York Heart Association classes III and IV), previous history of ischemic heart disease, history of cerebrovascular disease (ischemic stroke, transient ischemic attack or cerebral hemorrhage) or peripheral artery disease at the initial visit. CV events were considered as the primary outcome during follow-up: diagnoses of ischemic heart disease, cerebrovascular disease, or hospital admission for heart failure. The first CV event that occurred in each patient during the follow-up, which lasted until March 23, 2023, was considered. Events occurring after the first CV event were not considered for outcome definition. As the Valencia Community is part of the National Health System sharing a common, nationwide database, all cardiovascular events which occurred outside the Sagunto Hospital were captured in the same registry. Deaths were considered an event only if they were cardiovascular in origin. All cardiovascular outcomes were clinically reviewed by the principal investigator (ER). The study was approved by the Ethics Committee of the Hospital de Sagunto, and informed consent was not required, given the impossibility of requesting it a posteriori in this retrospective study.

### Statistical analysis and modeling process

First, we extracted 155 patient characteristics from the original registry before creating new variables (such as baseline data from electronic health records, comorbidities, physical examination measures, laboratory values, etc.), respecting the coding process to maintain patient anonymity and confidentiality and ensuring that it is not possible to identify them from the data provided. The database was then cleaned by eliminating values outside the correct range for numerical variables or categories with values not adjusted to the possible categories (S1 Text).

Given the algorithm’s ability to natively manage missing data, capture complex nonlinear interactions, and accommodate high-dimensional clinical variables, XGBoost was selected as the predictive model for this study. These properties are particularly relevant in structured electronic health record datasets, which typically combine heterogeneous variable types and derived longitudinal features. Furthermore, previous studies have demonstrated the strong performance of XGBoost and other gradient boosting approaches in predicting major adverse cardiovascular events in different clinical settings, including post-PCI STEMI cohorts and patients with coronary heart disease undergoing exercise evaluation [13,14].

Tree-based ensemble methods such as XGBoost are well suited for this context because they can automatically model higher-order interactions without imposing strict parametric assumptions. Moreover, gradient boosting incorporates regularization and shrinkage mechanisms that improve generalization performance in structured tabular data. In clinical prediction research, XGBoost and related boosting frameworks have shown robust discriminative performance in moderate-sized electronic health record datasets, with reported AUROC values typically ranging between 0.70 and 0.88 across diverse cardiovascular and critical care prediction tasks [15–19].

Index time and data availability. Baseline (index time) was defined as each participant’s first eligible visit in the Hypertension and Vascular Risk Unit. All predictors were constructed using only information recorded after baseline and available before outcome occurrence (cases) or before administrative censoring (non-cases), with no post-event data used for feature derivation.

To construct the predictive model, we first generated longitudinal summary features from the original variables to capture individual temporal patterns. For continuous variables, we computed descriptive statistics for each patient’s time series, including minimum, maximum, mean, standard deviation, and the difference between the first and last recorded values. These summaries were designed to characterize both variability and directionality of change over time.

To further explore structural relationships among these derived numerical features, we applied Principal Component Analysis (PCA). This dimensionality reduction step was performed both globally (including all derived variables together) and within subsets grouped by statistical descriptor (e.g., all means, all standard deviations), allowing us to identify latent patterns and reduce redundancy without incorporating outcome information.

For categorical variables, longitudinal trajectories were modeled using Latent Class Analysis (LCA). To represent temporal evolution, four equidistant observations were extracted from each individual time series: baseline, the 33rd percentile, the 66th percentile, and the last available measurement (S1 Fig). These time points were defined within each participant’s observed follow-up window. LCA enabled the identification of latent subgroups of patients sharing similar categorical evolution patterns.

Data completeness was high: 424 variables contained no missing values, and only five variables presented minimal missingness (three missing values each), totaling 15 missing observations across the cohort. All summary statistics were computed using available case data only. Both PCA and LCA were implemented as fully unsupervised procedures and were performed without access to outcome labels.

After feature engineering, the final dataset was divided into training (70%) and internal validation (30%) subsets using outcome-stratified sampling to preserve class distribution. All remaining missing values were confined to the training set; the validation set contained no missing data. Continuous variables with missing values in the training set were imputed using mean substitution calculated exclusively within the training data, thereby preventing information leakage.

The resulting principal components and latent class assignments were then incorporated as structured input features into the XGBoost model, a strategy that has been previously adopted in related machine learning studies to combine dimensionality reduction or pattern discovery techniques with tree-based algorithms, allowing the exploitation of nonlinear interactions while mitigating dimensionality and collinearity issues [20–22].

Although the present analysis was designed as a cumulative risk modeling framework, the same feature extraction strategy could be recalculated as new visits accrue, allowing dynamic risk updating. However, formal evaluation of visit-updated predictive performance would require a dedicated landmarking or time-dependent validation design.

For internal validation, we generated two mutually exclusive datasets with a balanced data distribution: 70% and 30% as the training and internal validation sets, respectively [23,24]. This partition was performed using stratified sampling based on the outcome variable, with the aim of preserving similar proportions of events and non-events in both subsets. Continuous variables are presented as mean ± standard deviation (SD) and compared between groups using the t-test, or as median (interquartile range [IQR]) and compared using the Mann-Whitney tests, depending on whether the data had a normal distribution or not. Categorical variables are presented as frequencies (percentages) and compared using the chi-square test.

Finally, hyperparameter tuning of the XGBoost model was performed using a Bayesian optimization approach combined with internal cross-validation. The optimization process targeted the main model hyperparameters, including the learning rate (eta), maximum tree depth, minimum child weight, subsample ratio, column subsample ratio, and the minimum loss reduction required to make a further partition (gamma).

Model performance during optimization was evaluated using 5-fold cross-validation on the training dataset, with the area under the receiver operating characteristic curve (AUC) and PR-AUC as the objectives functions. A maximum of 150 boosting rounds was allowed, and early stopping was applied to prevent overfitting.

The Bayesian optimization procedure consisted of an initial random exploration phase followed by an iterative search guided by an upper confidence bound acquisition function, allowing an efficient exploration of the hyperparameter space and selection of the optimal parameter combination. The final model was trained using the optimal hyperparameters identified through this process and subsequently evaluated on the internal validation dataset.

To address the imbalance between event and non-event cases, class weighting was incorporated in XGBoost by including the scale_pos_weight parameter, defined as the ratio between negative and positive cases in the training set (n_neg/ n_pos). This parameter adjusts the loss function by increasing the penalty for misclassification of the minority class without modifying the underlying outcome distribution. Model performance under class weighting was evaluated as a sensitivity analysis, with detailed results reported in S6 Table.

### Model evaluation and validation

We use a confusion matrix as a tool to evaluate the performance of the ranking algorithm, (S1 Table). This matrix functions as a contingency table and presents the expected results along with the actual statuses of the instances. It shows the number of patients correctly classified as healthy (no CVD events during follow-up, true positives, TP), those incorrectly classified as sick (with CVD events during follow-up) when they were healthy (false negatives, FNs), those misclassified as healthy when they were sick (false positives, FPs), and those correctly classified as sick (true negatives, TN). This confusion matrix allows for the calculation of various metrics, evaluates the performance of various models, compares them, and selects the optimal model (S2 Table). Sensitivity and specificity quantify how well the model identifies events and non-events, respectively. PPV and NPV indicate the proportion of correct positive and negative predictions. Prevalence reflects the event rate in the dataset, while detection rate and detection prevalence summarize the proportion of correctly detected events and the proportion predicted as events. Balanced accuracy combines sensitivity and specificity and is useful under class imbalance.

After obtaining the individual risks predicted with the XGBoost model, we divided the patients into two groups: those with CV events and those without CV events, based on the cut-off value determined by the receiver operating characteristic (ROC).

A calendar-period sensitivity analysis was conducted to assess temporal robustness (S4 Table). Full details of this analysis are provided in the Supplementary Material.

To contextualize the predictive performance of the selected model, baseline models using logistic regression and Random Forest were developed using the same predictor set and identical training/validation splits.

We calculated the range of importance of the selected variables using the SHAP values, which that allowed us to identify the variables that exerted the greatest influence and their impact on the predictive variable. These SHAP-based estimates are intended to support model-level interpretation of derived features summarizing longitudinal information, rather than causal inference or visit-level interpretation of individual raw measurements. Statistical analysis was performed using software R 4.3.3 (2024-02-29 ucrt).

To assess the longitudinal coherence of the predicted risk score in the presence of variable follow-up and censoring, Kaplan–Meier survival curves were constructed stratifying patients according to predicted risk categories (high vs. low risk based on the ROC-derived cut-off). Differences between groups were evaluated using the log-rank test. These curves are provided in the Supplementary Material (S5 Fig).

### Comparison with SCORE2

Using the SCORE2 algorithm, the CV risk at the first visit of patients who experienced a CV event was also calculated, and the proportion of high risk subjects was identified and compared with the results of the XGBoost model. It should be noted that SCORE2 provides a baseline 10-year risk estimate based on a predefined and limited set of variables, whereas the XGBoost model incorporates longitudinal follow-up-derived features and predicts the occurrence of an event during the observed follow-up period. Therefore, this comparison should be interpreted as a pragmatic benchmarking exercise within the same cohort rather than as a strictly equivalent methodological validation.

## Results

### Characteristics of the total cohort and training and validation cohorts

Of the 8,365 consecutive patients referred to a hospital’s hypertension unit between 13/March/1991 and 23/March/2023, a total of 3,588 met the inclusion criteria. Loss to follow-up, defined as complete loss of digital traceability, was minimal, with only 17 patients for whom follow-up could not be completed, including through national registries. As shown in Table 1, the mean age was 54.2 ± 1 years, 53% were women and 22.9% were smokers. The mean BP was 141/83 mmHg and the heart rate was 72 lpm. Except for weight and the proportion of smokers, all other clinically relevant variables (age, sex, BP, heart rate, glucose, cholesterol, estimated glomerular filtration rate and urinary albumin excretion) differed significantly between the group of patients free of events and those who developed CV events during follow-up (Table 1).

**Table 1:** Baseline characteristics of the study population according to the incidence of major cardiovascular events during the follow-up.

Variable	Total	No_events	Events	pvalue
Age (years)	54.2 ± 14.1	53.1 ± 13.9	62.4 ± 12.9	<0.05
Gender (f (%))	1890 (53%)	1693 (54%)	197 (45%)	<0.05
Smoker (y (%))	669 (22.9%)	593 (23.2%)	76 (21.0%)	0.370
Weight (kg)	79.1 ± 15.8	79 ± 15.9	79.7 ± 15.4	0.410
BMI (kg/m2)	29.9 ± 5.1	29.8 ± 5.2	30.6 ± 5	<0.05
SBP (mmHg)	141.1 ± 20.5	140.6 ± 19.6	144.5 ± 26.4	<0.05
DBP (mmHg)	82.7 ± 12.2	82.9 ± 11.9	81.6 ± 14.1	0.06
Heart rate (bpm)	71.7 ± 20.9	72.2 ± 20.6	67.9 ± 22.8	<0.05
eGFR (ml/min/1.73m2)	76.2 ± 20.6	77.4 ± 20.6	67.4 ± 19.2	<0.05
UACR (mg/g)*	8.0 (4.0-21.0)	8.0 (4.0-19.0)	14.0 (5.1-52.2)	<0.05
Glucose (mg/dl)	109.5 ± 30.6	107.7 ± 28.4	122.9 ± 40.6	<0.05
Cholesterol (mg/dl)	207 ± 39.7	206.7 ± 39.5	208.8 ± 41.1	0.499

*Median (IQR); BMI: Body mass index; SBP: Systolic blood pressure; DBP: Diastolic blood pressure; eGFR: estimated glomerular filtration rate; UACR: urinary albumin excretion rate.

The full cohort was randomly divided into the training (70% of participants) and validation (30% of participants) cohorts (Table 2). There were no significant differences in the key baseline variables between the training and the validation cohorts.

**Table 2:** Baseline characteristics of the study population according to belonging to the Training or the Validation cohort.

Variable	Total (n = 3,588)	Training	Validation	p value
Age (years)	54.2 ± 14.1	54.2 ± 14.1	54.3 ± 14.2	0.934
Gender (f (%))	1890 (53%)	1256 (53%)	634 (53%)	0.804
Smoker (n (%))	669 (22.9%)	451 (23.2%)	218 (22.3%)	0.683
Weight (kg)	79.1 ± 15.8	79 ± 15.6	79.4 ± 16.3	0.473
BMI (kg/m2)	29.9 ± 5.1	29.8 ± 5	30 ± 5.4	0.339
SBP (mmHg)	141.1 ± 20.5	140.7 ± 20.2	141.9 ± 21.2	0.124
DBP (mmHg)	82.7 ± 12.2	82.5 ± 12.2	83.1 ± 12.1	0.144
Heart rate (bpm)	71.7 ± 20.9	71.5 ± 21	72.1 ± 20.7	0.480
eGFR (ml/min/1.73m2)	76.2 ± 20.6	76.3 ± 20.8	76 ± 20.4	0.659
UACR (mg/g)*	8.0 (4.0-21.0)	8.0 (4.0-20.0)	9.0 (4.5-23.0)	0.329
Glucose (mg/dl)	109.5 ± 30.6	109.3 ± 30.8	110 ± 30.2	0.489
Cholesterol (mg/dl)	207 ± 39.7	207.8 ± 39.8	205.4 ± 39.5	0.790

*Median (IQR); BMI: Body mass index; SBP: Systolic blood pressure; DBP: Diastolic blood pressure; eGFR: estimated glomerular filtration rate; UACR: uriinary albumin excretion rate.

A total of 498 CV events were recorded during the study. Ischemic heart disease was the most common (n = 198: 75 acute myocardial infarction, 123 angina pectoris/stenting), followed by cerebrovascular disease (n = 163: 109 ischemic stroke, 18 hemorrhagic stroke, 36 transitory ischemic attack) and heart failure (n = 137). 85 suffered two or more events. The total number of CV deaths was 37. Only the first event was used to calculate the rate of CV events. The average follow-up time was 8.3 years, yielding an incidence rate of CV events of 1.93 (95% CI: 1.78-2.09) per 100 patient-years.

### ROC AUC, sensitivity, and specificity for prediction of the occurrence of future CV events of diverse AI models

**Fig 1:**
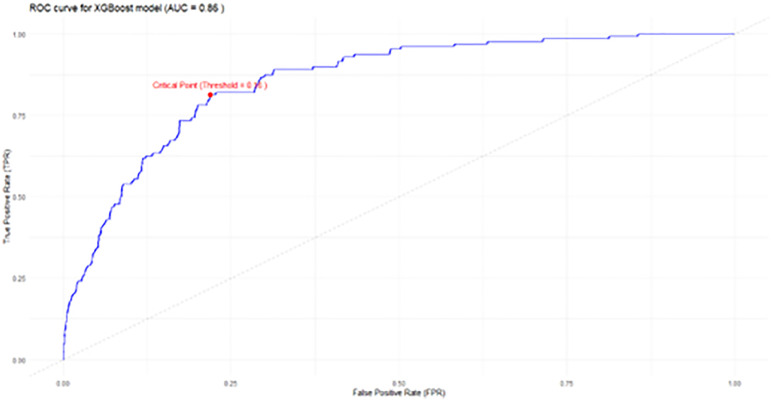
ROC curve for determination of the optimal cut-off point for prediction of new onset CVD.

After applying data imputation techniques to the selected variables and using principal component analysis (PCA) and Latent Class Analysis for polytomic variables (poLCA), the first model was developed, using the XGBoost method. From this, the 30 most influential variables were observed according to their absolute value calculated using the SHAP technique. Once these variables have been selected, a new XGBoost model was developed. This model was trained with the training cohort and evaluated with the validation cohort, to determine the optimal cutoff point using the ROC curve (Fig 1).

### XGBoost-x model for prediction of future CVD events in the validation cohort

Once the optimal cut-off point was obtained, we created a contingency table for the validation database. The contingency Table 3 illustrates the predicted CVD events versus the actual events.

**Table 3:** Contingency table.

Predicted CVD events	Real CVD events		
		No CVD events	CVD events
No CVD events	833	24
CVD events	235	104

**Fig 2:**
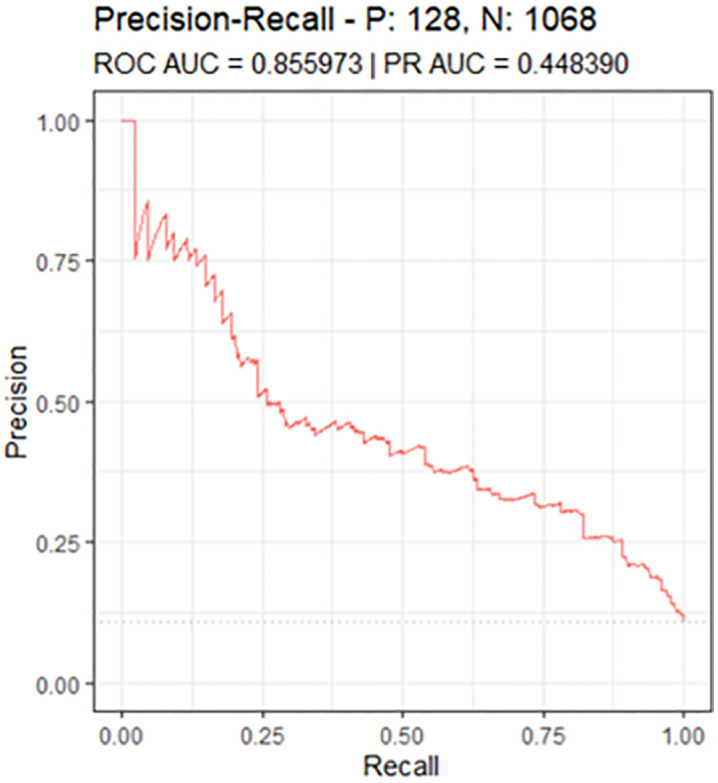
Precision–Recall (PR) curve in internal validation.

The model demonstrated good discriminative performance, with an area under the receiver operating characteristic curve (ROC AUC) of 0.856 in the validation cohort (Fig 2). At the selected optimal cut-off point, the overall accuracy was 0.78 (95% CI: 0.76–0.81), with a Kappa statistic of 0.34. Table 4 summarizes the remaining performance metrics.

**Table 4:** Model Performance Metrics.

Metric	Valor
Sensitivity	0.813
Specificity	0.780
Pos. Pred. Value	0.307
Neg. Pred. Value	0.972
Prevalence	0.107
Detection Rate	0.087
Detection Prevalence	0.283
Balanced Accuracy	0.796

To provide a more comprehensive evaluation of model performance, additional analyses assessing clinical utility (Decision Curve Analysis), and risk stratification capacity (cumulative gain or lift curve) are presented in S2 Fig, S3 Fig and S4 Fig, respectively.

The cumulative gain analysis specifically evaluated the concentration of observed events across increasing percentiles of predicted risk (S3 Table).

The results of the calendar-period sensitivity analysis are summarized in S4 Table.

Kaplan–Meier curves stratified by predicted risk demonstrated a significant separation between risk groups, with lower event-free survival in the high-risk category (log-rank p = 0.0001) (S5 Fig).

Comparative benchmarking against logistic regression and Random Forest models is summarized in S5 Table.

### Model explainability

The four most influential variables in the model were the number of antihypertensive drugs, need for antiplatelet medication, glomerular filtration rate, and the minimum LDL cholesterol value. It is important to clarify that antiplatelet medication does not reflect the occurrence of a CV event, but follows the recommended treatment of subclinical atherosclerosis/atherosclerosis, according to clinical Guidelines [4]. The most relevant, with high values significantly boosting the model’s predictions, was the maximum value of antihypertensive drugs, suggesting that this characteristic was a strong predictor of an increase in the value of CV events. Similarly, antiplatelet therapy and mean antihypertensive drugs also increased predictions when their values were high, although with a somewhat smaller impact. Glomerular filtration rate and minimal LDL cholesterol levels were less influential than the other two. High renal function values tended to increase event predictions, while low LDL cholesterol values reduced them, indicating an inverse relationship between LDL level and event prediction.

**Fig 3:**
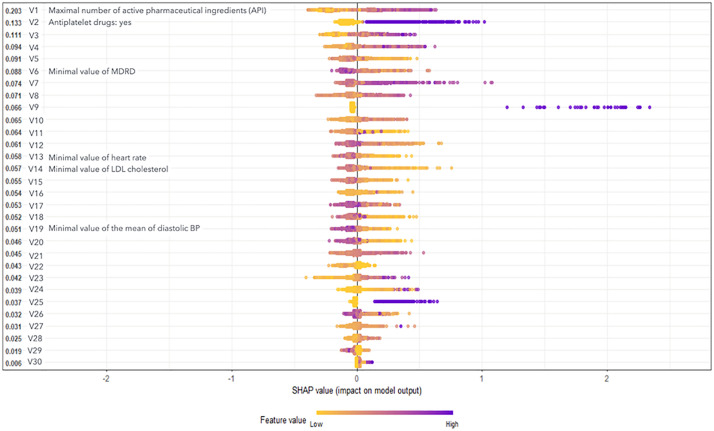
Graphical representation of the relative weight of the variables included in the XGBoost model. On the Y axis, the variables are ordered according to the forecast value from highest to lowest, on the X axis the color indicates the impact on the model (high, violet, low, yellow). Only variables discussed in Methods and easily understandable are included.

It is important to note that, as described in the Methods section, the variables that obtain significance represent complex constructions from the raw original variables in most cases. While the latter is intuitively understandable, the former responds to mathematical calculations that are difficult to describe. By grouping all 30 variables according to how they affect the model (Fig 3), and focusing on those that are clinically intuitively understandable, we identified three main groups. First, the variables with a strong positive impact on the prediction of events when their values are high, where the number of antihypertensive drugs, glomerular filtration rate, the presence of antiplatelet therapy and the LDL cholesterol value stand out among the easily understandable variables. In the second group, we found those with a moderate positive impact, where high values also slightly increase the prediction of the cardiac events. This includes variables that are mostly of a hemodynamic nature such as heart rate or blood pressure. Finally, there was a group of variables whose impact on the prediction was slight or almost negligible.

### Comparison of model performance with SCORE2

**Fig 4:**
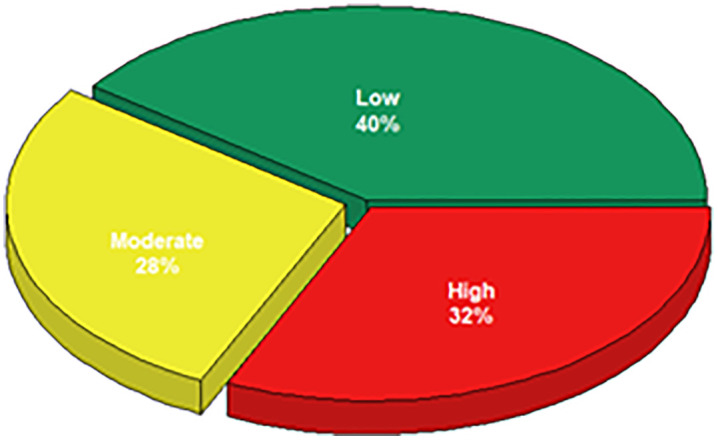
CV risk stratification at baseline according to SCORE2 in patients who suffered a CV event during the study.

Fig 4 includes all patients who suffered a CV event at follow-up and shows that the proportion of those classified as high-risk according to the SCORE2 algorithm reached 32%.

**Fig 5:**
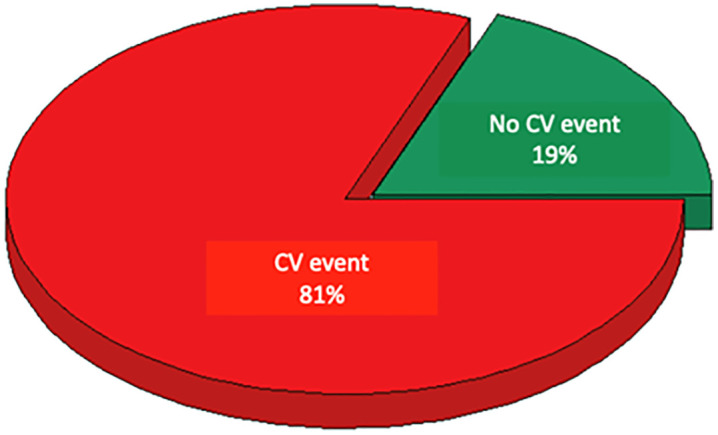
Proportion of patients in whom the XGBoost model predicted a CV event among those who experienced an event during the study.

Fig 5 represents the same patients as Fig 4, showing that the proportion of subjects in whom the XGBoost model predicted an event was 81%.

## Discussion

The main results of this study were, on the one hand, an algorithm with predictive capacity of CV events obtained by means of Artificial Intelligence (AI) techniques in a group of hypertensive subjects without previous events with significant accuracy in a real-life framework. Second, the identification of antihypertensive treatment, that is, the number of active ingredients prescribed and measured at each visit during follow-up (from baseline to the first CV event in cases, or to end of follow-up in censored participants), within the framework of repeated visits, as a variable with the highest predictive value.

Regarding the first result, it should be noted that the development of Framingham risk factors to predict CV events in the middle of the last century [25] represented a great advance in the management of CV diseases, since until that date the high prevalence of AMI and stroke was perceived as a fatalistic and inevitable event [26]. The identification of risk factors with predictive capacity has also allowed the development of increasingly precise risk tables in Europe that include a few significant variables, such as age, sex, systolic BP, total cholesterol and smoking habits in the most commonly used tables, SCORE [27], to predict fatal CV events over a 5-year time span. Subsequent studies have attempted to improve this precision by modifying the outcome to fatal or non-fatal CV events in 10 years (SCORE2 [28]), or extending the study population to older age ranges (SCORE2-OP [29]), or even including the diabetic population (SCORE2-DM [30]). In the first table, the number of five variables has been maintained, replacing only the total COL with the LDL-COL, the SCORE2-DM table incorporates three new variables that make it impossible to understand the graph directly and intuitively, and that requires the use of algorithms [31].

However, the residual risk after applying these tables remains high; thus, numerically and in absolute terms more CV events occur in the group of patients considered to have low and moderate CV risk than in the group with high CV risk [32,33].

The limited prognostic capacity of risk tables is partly due to the impossibility of collecting a large amount of clinical information generated in a medical visit in a simple table of five variables. AI has emerged in recent years as a technique capable of automatically processing large amounts of complex data using highly sophisticated statistical formulas to generate diagnostic and treatment algorithms, as well as in the field of CV diseases [34]. The vast majority of its applications are limited to cardiac imaging procedures [35], ECG [36], continuous monitoring of hemodynamic variables at the bedside [37], wearables [38] and genetics [39], but very few aim to improve the prediction of CV events, including CV mortality, from a clinical perspective based on electronic Health Records (EHR).

Several algorithms have been published in patients admitted to the ICU [40,41] or in the Emergency Department [42]. They achieved an accuracy of approximately 80% in predicting short-term mortality. Another goal of AI is to predict the incidence HTN [43]. However, very few algorithms have been specifically designed to improve the predictive capacity of CV events in the general or at-risk population from EHRs. The use of 1000 variables improved the prediction of CV events at 10 years among 109,490 individuals, with a precision of approximately 80% [44]. A similar result was obtained in the prediction of CV events in a population of 6,459 subjects free of atherosclerotic disease in the MESA study over an observation period of 13 years with its own algorithm (sensitivity: 0.86, specificity: 0.95, and AUC 0.92) compared to the traditional algorithm proposed by the ACC/AHA Risk Calculator (sensitivity: 0.76, specificity: 0.56, and AUC 0.71) [45]. Our study included fewer patients and fewer variables. However, the analysis of 3,588 patients followed for a mean of >8 years assessing 30 variables selected out of 155, surpasses the analytical capacity of traditional statistical programs, hence the need for AI. In similar studies with a small numbers of patients, Ren et al. [46] reported the development of an AI algorithm to predict CV events in 890 patients with diabetic nephropathy based on seven clinical variables extracted from their EHRs, with an AUC of 0.780. Brester et al. [47] improved with two different models (model 1: AUC 80.1%, model 2: AUC 71.6%), obtained by AI, the prediction of CV events in 2,682 men, (6.0% and 5.5%, respectively) with respect to the results derived from logistic regression with variables included manually.

More recently, several studies have been published that specifically examined the predictive ability of AI derived models in the hypertensive population. Lee et al. showed the superiority of the method Deep Neural Network over logistic regression in predicting CVD hospitalization and CVS death in 2,037,027 hypertensive subjects identified in the database of the Korean National Health Insurance Service one year after the last electronically registered visit [9]. In 259,873 newly diagnosed hypertensive patients, identified from administrative health data in Alberta, four different models derived from Machine learning methods provided similar personalized survival prediction of hospitalizations for CV events over a follow-up period of 3.8 years [10]. Similarly, a machine-learning model developed with electronic data in 2,781 hypertensive patients from the Chinese National Health and Nutrition Examination Survey proved to be efficient to predict CV disease [11]. The three studies share the ability of AI to identify hypertensive patients at high risk for CV events and so to facilitate integrated clinical workflows with higher accuracy, compared to conventional statistical methods.

Therefore, the magnitude of the precision obtained in our analysis (sensitivity, 81.3%; specificity; 78.0%) is comparable to the studies cited. A recent review of the quality of the results obtained in publications that used AI in the field of hypertension confirmed that the mean AUC was between 0.766 and 1.00 [48]. Differences in the key variables finally identified for constructing the prediction models may be due to differences in the follow-up time, presence of previous events and the availability and selection of clinical variables and outcomes.

It is important to note that, compared to SCORE2, the proportion of patients with CV events identified by XGBoost was much higher (81% versus 32%). This difference highlights potentially meaningful implications for risk classification in hypertensive patients, as it underlines not only the need for improvement in performance of the most widely used and most recent algorithms in daily clinical practice, such as SCORE2, but also multiplies almost three times the accuracy of the model we present. However, this difference must be interpreted with caution, as SCORE2 is a baseline, fixed-horizon risk estimation tool, whereas our model integrates longitudinal information accumulated during follow-up and predicts a non-horizon-specific event outcome. Thus, the two approaches are not directly equivalent in terms of prediction framework or information set. Despite this fundamental, methodological difference, from a clinical point of view we considered that comparing the new developed algorithm with SCORE2 is reasonable, because SCORE2 remains the recommended gold standard for risk stratification in hypertensive subjects in daily clinical practice, according to the most recent guidelines [4].

Regarding the second main result, that is, the identification of significant variables with predictive power in our analysis, the predominant role of antihypertensive drug treatment stands out compared to the marginal importance of blood pressure levels. This observation highlights two essential limitations that mark the fundamental difference between the traditional risk tables and AI-generated algorithms. First, the risk tables were designed to stratify subjects with low or moderate CV risk and with mild disease burden. However, they did not include the number of drugs used as a variable. Second, the conceptual framework of the risk tables does not include repeated measures. Similarly, traditional logistic regression analyses rely mainly on single measures at a given time point, called “present BP” in the literature, to predict an outcome in terms of CV event at another time point, usually very distant.

The association between CV diseases and prolonged exposure to high blood pressure has been repeatedly analyzed through its quantification using indices such as the time-averaged BP [49], cumulative BP [50], BP trajectory patterns [51], and age of hypertension onset [52]. While these four indices can improve the prognosis of CV events, the difficulty in calculating them and their heterogeneity limit their implementation [53]; thus, their use in clinical practice has been practically non-existent until now.

The finding of the high predictive power of antihypertensive treatment in repeated measures in the long term may seem tautological at first glance and not surprising, but it underlines its importance as a main marker of exposure to arterial hypertension in a series of repeated measures. In our study, it may be a reflection of the greater burden of disease in hypertensive patients as well as a marker of the adequate use of the available antihypertensive arsenal, and therefore, justifies its role as the main variable.

Among the limitations of this study, first, the follow-up schedule was not protocolized, but reflected individualized clinical management, resulting in heterogeneous visit patterns across patients.

Second, the study period spans more than three decades, during which treatment guidelines and pharmacological strategies evolved, potentially introducing temporal heterogeneity in clinical management.

Third, the study population consisted exclusively of hypertensive patients referred to a specialized unit; therefore, external generalizability to broader or lower-risk populations may be limited.

Fourth, although XGBoost was compared with SCORE2, we did not stratify analyses according to SCORE2-OP or SCORE2-DM categories, which may have influenced the comparative results in specific subgroups.

Additionally, the primary endpoint was defined as the occurrence of a first cardiovascular event during the observed follow-up. Consequently, follow-up duration varied across individuals and censoring was present. The model therefore estimates accumulated risk over the available observation time rather than a fixed-horizon (e.g., 10-year) absolute risk. Predicted probabilities should not be interpreted as horizon-specific risk estimates, and direct numerical comparisons with SCORE2 should be interpreted with caution.

Furthermore, while SCORE2 is derived exclusively from baseline variables within a predefined risk framework, our model incorporates longitudinal follow-up-derived features, including treatment-related patterns. This fundamental difference in information structure limits strict methodological comparability between both approaches.

Besides, several predictors were constructed by summarizing repeated measurements up to the time of event (cases) or censoring (non-cases). For patients experiencing an event, only measurements recorded prior to the event date were included. Although no post-event data were used, this cumulative feature construction differs from a strictly baseline-only or fixed-horizon prospective prediction design. Landmarking strategies or formal time-to-event modeling would be required to fully evaluate horizon-specific or dynamically updated risk prediction.

In addition, comparisons with logistic regression and Random Forest should be interpreted as baseline benchmarking analyses, since a fully exhaustive head-to-head comparison would ideally require model-specific optimization pipelines and, potentially, model-specific feature engineering strategies.

Among the strengths of this study is that it was performed in daily clinical practice in the real world. Second, all the patients were treated in a single Hypertension Unit by the same medical team. Third, it had a sufficient number of patients and follow-up time to enable a robust statistical analysis.

## Conclusions

The prediction model developed using artificial intelligence (XGBoost) demonstrated a significant ability to predict cardiovascular events in hypertensive patients with no previous history of CVD, outperforming the SCORE2 algorithm. The variable with the highest predictive power was the number of antihypertensive drugs prescribed throughout the follow-up, which highlights the value of repeated treatment measures as an indirect marker of disease burden and prolonged exposure to hypertension, above and beyond single blood pressure measurements.

The integration of multiple clinical and biochemical variables, processed in a time series, improved risk stratification compared to traditional tables, limited by a reduced number of factors, and using specific measures. This approach offers the possibility of identifying patients with a higher cardiovascular risk earlier and more accurately, facilitating the implementation of personalized preventive strategies.

Taken together, these results support the progressive incorporation of AI algorithms into clinical practice as complementary tools to optimize the primary prevention of cardiovascular diseases.

## Supporting information

S1 Text Supplementary methods. (PDF)

S1 Table Contingency table. Development of CVD events was assessed during follow-up. a) Theoretical contingency matrix. b) Contingency matrix in the validation cohort.
(PDF)

S2 Table Mathematical definitions. (PDF)

S3 Table Event capture across top predicted-risk percentiles in internal validation. Cumulative gain summary showing the number and proportion of observed CVD events captured when selecting patients in the highest predicted-risk strata (top 5%, 10%, 20%, 30%, and 50%) ranked by the XGBoost-predicted probability.
(PDF)

S4 Table Discrimination performance (AUC) of the XGBoost model across calendar periods. (PDF)

S5 Table Comparative performance of Random Forest, Logistic Regression, and XGBoost in the internal validation cohort. (PDF)

S6 Table Confusion matrix and performance metrics of the XGBoost model with class weighting in the validation cohort (event prevalence: 10.7%). (PDF)

S1 Fig Example of a time series of a selected variable. (PDF)

S2 Fig Calibration plot in internal validation. (PDF)

S3 Fig Decision curve analysis in internal validation. Net benefit of using the XGBoost model across threshold probabilities compared with “treat-all” and “treat-none” strategies; higher net benefit indicates greater clinical utility at a given threshold.
(PDF)

S4 Fig Cumulative gain (lift) curve in internal validation. Cumulative proportion of observed CVD events captured as patients are ranked from highest to lowest XGBoost-predicted risk. The dashed diagonal indicates random selection; deviation above it reflects event enrichment in the highest-risk strata.
(PDF)

S5 Fig Kaplan–Meier curves of event-free survival stratified by predicted risk categories (low vs. high risk) according to the XGBoost model. Risk groups were defined using the ROC-derived optimal cut-off. Differences between groups were assessed using the log-rank test (p = 0.0001).
(PDF)

## References

[1] World Health Organization. Cardiovascular diseases (CVDs). https://www.who.int/news-room/fact-sheets/detail/cardiovascular-diseases-(cvds). 2021.

[2] Esteva A, Robicquet A, Ramsundar B, Kuleshov V, DePristo M, Chou K, et al. A guide to deep learning in healthcare. Nat Med. 2019;25(1):24–9. doi: 10.1038/s41591-018-0316-z 30617335

[3] Global Cardiovascular Risk Consortium. Global Effect of Modifiable Risk Factors on Cardiovascular Disease and Mortality. N Engl J Med. 2023;389(14):1273–85. doi: 10.1056/NEJMoa2206916 37632466 PMC10589462

[4] Mancia G, Kreutz R, Brunström M, Burnier M, Grassi G, Januszewicz A, et al. 2023 ESH Guidelines for the management of arterial hypertension The Task Force for the management of arterial hypertension of the European Society of Hypertension: Endorsed by the International Society of Hypertension (ISH) and the European Renal Association (ERA). J Hypertens. 2023;41(12):1874–2071. doi: 10.1097/HJH.0000000000003480 37345492

[5] Hannun AY, Rajpurkar P, Haghpanahi M, Tison GH, Bourn C, Turakhia MP, et al. Cardiologist-level arrhythmia detection and classification in ambulatory electrocardiograms using a deep neural network. Nat Med. 2019;25(1):65–9. doi: 10.1038/s41591-018-0268-3 30617320 PMC6784839

[6] Gulshan V, Peng L, Coram M, Stumpe MC, Wu D, Narayanaswamy A, et al. Development and Validation of a Deep Learning Algorithm for Detection of Diabetic Retinopathy in Retinal Fundus Photographs. JAMA. 2016;316(22):2402–10. doi: 10.1001/jama.2016.17216 27898976

[7] Chen S-F, Loguercio S, Chen K-Y, Lee SE, Park J-B, Liu S, et al. Artificial Intelligence for Risk Assessment on Primary Prevention of Coronary Artery Disease. Curr Cardiovasc Risk Rep. 2023;17(12):215–31. doi: 10.1007/s12170-023-00731-4

[8] Mitchell GF, Rong J, Larson MG, Korzinski TJ, Xanthakis V, Sigurdsson S. Vascular age assessed from an uncalibrated, noninvasive pressure waveform by using a deep learning approach: the AI-vascularage model. Hypertension. 2024;81(1):193–201. doi: 10.1161/HYPERTENSIONAHA.123.21638 37901957 PMC10842456

[9] Lee S-J, Lee S-H, Choi H-I, Lee J-Y, Jeong Y-W, Kang D-R, et al. Deep Learning Improves Prediction of Cardiovascular Disease-Related Mortality and Admission in Patients with Hypertension: Analysis of the Korean National Health Information Database. J Clin Med. 2022;11(22):6677. doi: 10.3390/jcm11226677 36431154 PMC9697313

[10] Wang M. Explainable machine-learning-based cardiovascular disease prediction in patients with hypertension: Algorithm comparison and SHapley Additive exPlanations (SHAP) analysis. Arch Cardiovasc Dis. 2026;119(4):273–82. doi: 10.1016/j.acvd.2025.09.005 41535161

[11] Feng Y, Leung AA, Lu X, Liang Z, Quan H, Walker RL. Personalized prediction of incident hospitalization for cardiovascular disease in patients with hypertension using machine learning. BMC Med Res Methodol. 2022;22(1):325. doi: 10.1186/s12874-022-01814-3 36528631 PMC9758895

[12] Ombuena P, Rodilla Sala E, Costa Muñoz JA, Pascual Izuel JM. Hipertensión arterial y prediabetes. Med Clin (Barc). 2016;147(9):387–92. doi: 10.1016/j.medcli.2016.06.032 27575529

[13] Zhang N, Wang J, Shen C, Pei Y, Li W, Wang H, et al. XGBoost based machine learning prediction model for major adverse cardiovascular events after PCI in STEMI patients. Sci Rep. 2026;16(1):4419. doi: 10.1038/s41598-025-34441-1 41484374 PMC12865028

[14] Shen T, Liu D, Lin Z, Ren C, Zhao W, Gao W. A Machine Learning Model to Predict Cardiovascular Events during Exercise Evaluation in Patients with Coronary Heart Disease. J Clin Med. 2022;11(20):6061. doi: 10.3390/jcm11206061 36294382 PMC9605581

[15] Chen T. XGBoost: A Scalable Tree Boosting System. Cornell University. 2016.

[16] Lundberg SM, Erion G, Chen H, DeGrave A, Prutkin JM, Nair B, et al. From Local Explanations to Global Understanding with Explainable AI for Trees. Nat Mach Intell. 2020;2(1):56–67. doi: 10.1038/s42256-019-0138-9 32607472 PMC7326367

[17] Song X, Shi J, Zhu C, Xian F, Dong Z, Li J. XGBoost machine learning algorithm for predicting unplanned readmission in elderly patients with coronary heart disease. Geriatr Nurs. 2025;66(Pt B):103609. doi: 10.1016/j.gerinurse.2025.103609 40945246

[18] Hidayaturrohman QA, Hanada E. Impact of Data Pre-Processing Techniques on XGBoost Model Performance for Predicting All-Cause Readmission and Mortality Among Patients with Heart Failure. BioMedInformatics. 2024;4(4):2201–12. doi: 10.3390/biomedinformatics4040118

[19] Chen E, Fan S, Pishgar E, Alaei K, Placencia G, Pishgar M. XGBoost-based prediction of ICU mortality in sepsis-associated acute kidney injury patients using MIMIC-IV database with validation from eICU database. medRxiv. 2025;2025–02.

[20] Mochurad L, Babii V, Boliubash Y, Mochurad Y. Improving stroke risk prediction by integrating XGBoost, optimized principal component analysis, and explainable artificial intelligence. BMC Med Inform Decis Mak. 2025;25(1):63. doi: 10.1186/s12911-025-02894-z 39920691 PMC11806876

[21] Mukherjee M, Mukherjee S, Thokala HR, Ali RH. Classifying complex multimorbidity using latent class analysis and machine learning to generate insights into clustering of mental and cardiometabolic conditions. PLoS One. 2025;20(11):e0335676. doi: 10.1371/journal.pone.0335676 41248181 PMC12622840

[22] Lee JS, Lee SK. Identification of risk groups for and factors affecting metabolic syndrome in South Korean single-person households using latent class analysis and machine learning techniques: Secondary analysis study. JMIR Form Res. 2023. doi: 10.2196/42756 PMC10523223 37698907

[23] Cardiovascular disease prediction through machine learning: A comparative study of ensemble techniques. Revolutionary Advances in Computing and Electronics: An International Journal. 2026;1(1):27–40. doi: 10.65890/RACE.v1i1.2

[24] Ng BA. Comparative analysis of machine learning techniques for classifying the risk of cardiovascular diseases. Emerging advancements in AI and big data technologies in business and society. IGI Global. 2024:158–82.

[25] Dawber TR, Moore FE, Mann GV. Coronary heart disease in the Framingham study. Am J Public Health Nations Health. 1957;47(4 Pt 2):4–24. doi: 10.2105/ajph.47.4_pt_2.4 13411327 PMC1550985

[26] The Framingham Heart Study and the Epidemiology of Cardiovascular Diseases: A Historical Perspective. Lancet. 2014;383(9921):999–1008. doi: 10.1016/S0140-6736(13)61752-3 24084292 PMC4159698

[27] Conroy RM, Pyörälä K, Fitzgerald AP, Sans S, Menotti A, De Backer G, et al. Estimation of ten-year risk of fatal cardiovascular disease in Europe: the SCORE project. Eur Heart J. 2003;24(11):987–1003. doi: 10.1016/s0195-668x(03)00114-3 12788299

[28] SCORE2 working group and ESC Cardiovascular risk collaboration. SCORE2 risk prediction algorithms: new models to estimate 10-year risk of cardiovascular disease in Europe. Eur Heart J. 2021;42(25):2439–54. doi: 10.1093/eurheartj/ehab309 34120177 PMC8248998

[29] SCORE2-OP working group and ESC Cardiovascular risk collaboration. SCORE2-OP risk prediction algorithms: estimating incident cardiovascular event risk in older persons in four geographical risk regions. Eur Heart J. 2021;42(25):2455–67. doi: 10.1093/eurheartj/ehab312 34120185 PMC8248997

[30] SCORE2-Diabetes Working Group, ESC Cardiovascular Risk Collaboration. 30 SCORE2-Diabetes: 10-year cardiovascular risk estimation in type 2 diabetes in Europe. European Heart Journal. 2023;44:2544–56. doi: 10.1093/eurheartj/ehad260 37247330 PMC10361012

[31] Score2 Diabetes Calculator. https://www.mdcalc.com/calc/10510/score2-diabetes

[32] Akosah KO, Schaper A, Cogbill C, Schoenfeld P. Preventing myocardial infarction in the young adult in the first place: how do the National Cholesterol Education Panel III guidelines perform? J Am Coll Cardiol. 2003;41(9):1475–9. doi: 10.1016/s0735-1097(03)00187-6 12742284

[33] Costa JA, Rodilla E, Cardona J, González C, Pascual JM. Síndrome metabólico y complicaciones cardiovasculares en el paciente hipertenso. Med Clin (Barc). 2012;139(4):150–6. doi: 10.1016/j.medcli.2011.05.018 21813141

[34] Armoundas AA, Narayan SM, Arnett DK, Spector-Bagdady K, Bennett DA, Celi LA, et al. Circulation. 2024;149(14):e1028–50. doi: 10.1161/CIR.0000000000001201 38415358 PMC11042786

[35] Wu JT-Y, de la Hoz MÁA, Kuo P-C, Paguio JA, Yao JS, Dee EC, et al. Developing and Validating Multi-Modal Models for Mortality Prediction in COVID-19 Patients: a Multi-center Retrospective Study. J Digit Imaging. 2022;35(6):1514–29. doi: 10.1007/s10278-022-00674-z 35789446 PMC9255527

[36] Karri R, Kawai A, Thong YJ, Ramson DM, Perry LA, Segal R, et al. Machine Learning Outperforms Existing Clinical Scoring Tools in the Prediction of Postoperative Atrial Fibrillation During Intensive Care Unit Admission After Cardiac Surgery. Heart Lung Circ. 2021;30(12):1929–37. doi: 10.1016/j.hlc.2021.05.101 34215511

[37] Bollepalli SC, Sevakula RK, Au-Yeung W-TM, Kassab MB, Merchant FM, Bazoukis G, et al. Real-Time Arrhythmia Detection Using Hybrid Convolutional Neural Networks. J Am Heart Assoc. 2021;10(23):e023222. doi: 10.1161/JAHA.121.023222 34854319 PMC9075394

[38] Bayoumy K, Gaber M, Elshafeey A, Mhaimeed O, Dineen EH, Marvel FA, et al. Smart wearable devices in cardiovascular care: where we are and how to move forward. Nat Rev Cardiol. 2021;18(8):581–99. doi: 10.1038/s41569-021-00522-7 33664502 PMC7931503

[39] Wang K, Zhang H, Kugathasan S, Annese V, Bradfield JP, Russell RK, et al. Diverse genome-wide association studies associate the IL12/IL23 pathway with Crohn Disease. Am J Hum Genet. 2009;84(3):399–405. doi: 10.1016/j.ajhg.2009.01.026 19249008 PMC2668006

[40] Pirracchio R, Petersen ML, Carone M, Rigon MR, Chevret S, van der Laan MJ. Mortality prediction in intensive care units with the Super ICU Learner Algorithm (SICULA): a population-based study. Lancet Respir Med. 2015;3(1):42–52. doi: 10.1016/S2213-2600(14)70239-5 25466337 PMC4321691

[41] Holmgren G, Andersson P, Jakobsson A, Frigyesi A. Artificial neural networks improve and simplify intensive care mortality prognostication: a national cohort study of 217,289 first-time intensive care unit admissions. J Intensive Care. 2019;7:44. doi: 10.1186/s40560-019-0393-1 31428430 PMC6697927

[42] Wu TT, Zheng RF, Lin ZZ, Gong HR, Li H. A machine learning model to predict critical care outcomes in patient with chest pain visiting the emergency department. BMC Emerg Med. 2021;21(1):112. doi: 10.1186/s12873-021-00501-8 34620086 PMC8496015

[43] Ye C, Fu T, Hao S, Zhang Y, Wang O, Jin B, et al. Prediction of Incident Hypertension Within the Next Year: Prospective Study Using Statewide Electronic Health Records and Machine Learning. J Med Internet Res. 2018;20(1):e22. doi: 10.2196/jmir.9268 29382633 PMC5811646

[44] Zhao J, Feng Q, Wu P, Lupu RA, Wilke RA, Wells QS, et al. Learning from Longitudinal Data in Electronic Health Record and Genetic Data to Improve Cardiovascular Event Prediction. Sci Rep. 2019;9(1):717. doi: 10.1038/s41598-018-36745-x 30679510 PMC6345960

[45] Kakadiaris IA, Vrigkas M, Yen AA, Kuznetsova T, Budoff M, Naghavi M. J Am Heart Assoc. 2018;7(22):e009476. doi: 10.1161/JAHA.118.009476 30571498 PMC6404456

[46] Ren J, Liu D, Li G, Duan J, Dong J, Liu Z. Prediction and Risk Stratification of Cardiovascular Disease in Diabetic Kidney Disease Patients. Front Cardiovasc Med. 2022;9:923549. doi: 10.3389/fcvm.2022.923549 35811691 PMC9263287

[47] Brester C, Voutilainen A, Tuomainen T-P, Kauhanen J, Kolehmainen M. Epidemiological predictive modeling: lessons learned from the Kuopio ischemic heart disease risk factor study. Ann Epidemiol. 2022;70:1–8. doi: 10.1016/j.annepidem.2022.03.010 35354081

[48] Silva GFS, Fagundes TP, Teixeira BC, Chiavegatto Filho ADP. Machine Learning for Hypertension Prediction: a Systematic Review. Curr Hypertens Rep. 2022;24(11):523–33. doi: 10.1007/s11906-022-01212-6 35731335

[49] Ayala Solares JR, Canoy D, Raimondi FED, Zhu Y, Hassaine A, Salimi-Khorshidi G, et al. Long-Term Exposure to Elevated Systolic Blood Pressure in Predicting Incident Cardiovascular Disease: Evidence From Large-Scale Routine Electronic Health Records. J Am Heart Assoc. 2019;8(12):e012129. doi: 10.1161/JAHA.119.012129 31164039 PMC6645648

[50] Pool LR, Ning H, Wilkins J, Lloyd-Jones DM, Allen NB. Use of Long-term Cumulative Blood Pressure in Cardiovascular Risk Prediction Models. JAMA Cardiol. 2018;3(11):1096–100. doi: 10.1001/jamacardio.2018.2763 30193291 PMC6583053

[51] Tielemans SMAJ, Geleijnse JM, Laughlin GA, Boshuizen HC, Barrett-Connor E, Kromhout D. Blood pressure trajectories in relation to cardiovascular mortality: The Rancho Bernardo Study. J Hum Hypertens. 2017;31(8):515–9. doi: 10.1038/jhh.2017.20 28332507

[52] Niiranen TJ, McCabe EL, Larson MG, Henglin M, Lakdawala NK, Vasan RS, et al. Heritability and risks associated with early onset hypertension: multigenerational, prospective analysis in the Framingham Heart Study. BMJ. 2017;357:j1949. doi: 10.1136/bmj.j1949 28500036 PMC5430541

[53] Nuotio J, Suvila K, Cheng S, Langén V, Niiranen T. Longitudinal blood pressure patterns and cardiovascular disease risk. Ann Med. 2020;52(3–4):43–54. doi: 10.1080/07853890.2020.1733648 32077328 PMC7877994

