# Supplementary material for: Optimization of artificial intelligence models for prediction of new-onset cardiovascular disease in patients with arterial hypertension
Source: PLOS Digit Health. 2026 May 21;5(5):e0001441. doi: 10.1371/journal.pdig.0001441 (PMC13193449; doi:10.1371/journal.pdig.0001441)
Supplement: S1 Text — (PDF) [file pdig.0001441.s001.pdf]

Supplementary methods

Variable selection

Of the 155 variables contained in each visit, 37 were discarded, either because they did not provide significant data for the study (e.g., zip code, city), because the quality of the variable was not acceptable or because most of the cases had missing values, data imputation techniques could not be applied. For the remainder of the study, we applied data imputation techniques. The remaining variables were divided into two groups to create new variables based on the type of data, numerical and categorical variables. With the numerical variables, we initially create different statistical values (minimum, mean, standard deviation, maximum, and the difference between the first and last shots) for each time series for each variable. Once this is done, from the created variables, we used the principal component analysis (PCA) technique to achieve new relationships between the variables. Second, we analyzed categorical variables with the aim of identifying internal patterns within each individual and, in this way, provided added value to the study. To address this type of variable, we applied advanced statistical techniques such as Latent Class Analysis (LCA) or its variant for polytomic variables, known as Polytomous Variable Latent Class Analysis (poLCA). These methodologies allow individuals to be grouped into latent classes that are not directly observable, based on their responses to a set of categorical variables. The primary purpose of using poLCA or LCA is to identify subsets of individuals with similar profiles, which is especially useful in studies in which differences between categories are not apparent to the naked eye. For this study we selected four shots of the time series of equidistant variables. That is, the first was located in the 33rd percentile, followed by the one located in the 66th percentile and the last in the time series (Supplementary Figure 1). The first model was created with these new variables (either numerical or categorical). We selected the 30 variables that had the greatest influence when creating the predictive model, using the SHAP value technique. SHapley Additive

exPlanations (SHAP) values can be used to interpret the impact of each characteristic on the prediction model. If a feature has a high positive contribution, it implies that its high presence or value is associated with a higher probability that the model will predict the class in question. Conversely, if a feature has a high negative contribution, it means that its presence is associated with a lower probability that the model will predict the class in question. Therefore we attempted to calculate the SHAP values for each variable and observation, which reflect the individual contribution of each variable to the model's prediction. The global importance of each variable was then determined by calculating the average absolute value of these SHAP values for all observations. The variables were ordered by their importance, and the 30 with the highest average absolute values were selected, thus ensuring a fair selection based on their actual impact on the model's predictions.

Model Performance

After the trait selection process, these 30 variables were used as independent predictors using the XGBoost method to build the predictive model and the Bayesian optimizer to improve the results. In the optimization process we used the hyperparameters, learning rate, maximum depth of the trees, minimum weight of the sum of the values of the second derivative of the loss function in a daughter partition, minimum reduction of the required loss (gamma), proportion of samples and fraction of features (columns) that were randomly used in each tree construction. Each of these hyperparameters was assigned a range of possible values. Once optimized, the model was trained using the optimal hyperparameters and tested with the validation data.

We used the ROC curve to determine the optimal cut-off point in the predictive model because it allows us to balance sensitivity and specificity, according to the needs of the problem. From this cut-off point, we create the classes of predicted values, classifying the cases as cardiovascular events or non-cardiovascular events, thus optimizing the

performance of the model based on the cost-benefit ratio of the classification errors. In Figure 1 we can see the ROC curve and optimal cut-off point.
