# Supplementary material for: Optimization of artificial intelligence models for prediction of new-onset cardiovascular disease in patients with arterial hypertension
Source: PLOS Digit Health. 2026 May 21;5(5):e0001441. doi: 10.1371/journal.pdig.0001441 (PMC13193449; doi:10.1371/journal.pdig.0001441)
Supplement: S1 Table — Development of CVD events was assessed during follow-up. a) Theoretical contingency matrix. b) Contingency matrix in the validation cohort. (PDF) [file pdig.0001441.s002.pdf]

S1 Table. Contingency table. Development of CVD events was assessed during follow-up.

a) Theoretical contingency matrix

		Actual events	
		No CVD events	CVD events
**Prediction**	No CVD events	**TP**	FP
	CVD events	FN	**TN**

b) Contingency matrix in the validation cohort

		Actual VCVD events	
		No CVD events	CVD events
**Predicted CVD events**	No CVD events	833	24
	CVD events	235	104
