# Supplementary material for: Optimization of artificial intelligence models for prediction of new-onset cardiovascular disease in patients with arterial hypertension
Source: PLOS Digit Health. 2026 May 21;5(5):e0001441. doi: 10.1371/journal.pdig.0001441 (PMC13193449; doi:10.1371/journal.pdig.0001441)
Supplement: S2 Table — (PDF) [file pdig.0001441.s003.pdf]

S2 Table: Mathematical definitions

- Precision or Positive predictive value (PPV): $\frac{\sum True\ positive}{\sum Test\ outcome\ positive}$
- Negative predictive value: $\frac{\sum True\ negative}{\sum Test\ outcome\ positive}$
- Specificity or True Negative Rate (TNR): $\frac{\sum True\ negative}{\sum Condition\ negative}$
- Sensitivity or True Positive Rate (TPR): $\frac{\sum True\ positive}{\sum Condition\ positive}$
- Positive likelihood ratio (LR+):
- Negative likelihood ratio (LR-): $1 - \frac{Sensitivity}{Specificity}$
- Accuracy: $\frac{\sum True\ positive + \sum True\ positive}{\sum Total\ population}$
- Area under the curve ROC (AUC): The ROC curve, represents Sensitivity vs. (1-Specificity) at different classification thresholds (Figure 1).
