# Supplementary material for: Optimization of artificial intelligence models for prediction of new-onset cardiovascular disease in patients with arterial hypertension
Source: PLOS Digit Health. 2026 May 21;5(5):e0001441. doi: 10.1371/journal.pdig.0001441 (PMC13193449; doi:10.1371/journal.pdig.0001441)
Supplement: S3 Table — Cumulative gain summary showing the number and proportion of observed CVD events captured when selecting patients in the highest predicted-risk strata (top 5%, 10%, 20%, 30%, and 50%) ranked by the XGBoost-predicted probability. (PDF) [file pdig.0001441.s004.pdf]

S3 Table. Event capture across top predicted-risk percentiles in internal validation.

top_pct	cum_pop	events_captured_n	events_captured_pct
Top 5%	0.05	31	0.24
Top 10%	0.1	54	0.42
Top 20%	0.2	83	0.65
Top 30%	0.3	105	0.82
Top 50%	0.5	120	0.94

Cumulative gain summary showing the number and proportion of observed CVD events captured when selecting patients in the highest predicted-risk strata (top 5%, 10%, 20%, 30%, and 50%) ranked by the XGBoost-predicted probability.
