# Supplementary material for: Optimization of artificial intelligence models for prediction of new-onset cardiovascular disease in patients with arterial hypertension
Source: PLOS Digit Health. 2026 May 21;5(5):e0001441. doi: 10.1371/journal.pdig.0001441 (PMC13193449; doi:10.1371/journal.pdig.0001441)
Supplement: S4 Table — (PDF) [file pdig.0001441.s005.pdf]

S4 Table: Discrimination performance (AUC) of the XGBoost model across calendar periods.

Period	AUC	N	Events
1991-2007	0.82	887	116
2008-2016	0.89	1379	133
2017-2023	0.77	1322	189
