# Supplementary material for: Optimization of artificial intelligence models for prediction of new-onset cardiovascular disease in patients with arterial hypertension
Source: PLOS Digit Health. 2026 May 21;5(5):e0001441. doi: 10.1371/journal.pdig.0001441 (PMC13193449; doi:10.1371/journal.pdig.0001441)
Supplement: S5 Table — (PDF) [file pdig.0001441.s006.pdf]

**S5 Table. Comparative performance of Random Forest, Logistic Regression, and XGBoost in the internal validation cohort.**

| Metric | Random Forest | Logistic Regression | XGBoost |
|---|---|---|---|
| Accuracy | 0.86 | 0.80 | 0.78 |
| Sensitivity | 0.13 | 0.56 | 0.813 |
| Specificity | 0.98 | 0.84 | 0.780 |
| PPV | 0.60 | 0.37 | 0.307 |
| NPV | 0.87 | 0.92 | 0.972 |
