# Supplementary material for: Optimization of artificial intelligence models for prediction of new-onset cardiovascular disease in patients with arterial hypertension
Source: PLOS Digit Health. 2026 May 21;5(5):e0001441. doi: 10.1371/journal.pdig.0001441 (PMC13193449; doi:10.1371/journal.pdig.0001441)
Supplement: S6 Table — (PDF) [file pdig.0001441.s007.pdf]

S6 Table. Confusion matrix and performance metrics of the XGBoost model with class weighting in the validation cohort (event prevalence: 10.7%).

**Confusion Matrix (Validation Cohort)**	
Metric	Value
True Negatives (TN)	837
False Positives (FP)	32
False Negatives (FN)	231
True Positives (TP)	96
**Performance Metrics**	
Metric	Value
Accuracy	0.780
Sensitivity	0.750
Specificity	0.784
Positive Predictive Value	0.294
Negative Predictive Value	0.963
Prevalence	0.107
Balanced Accuracy	0.767
