# Supplementary figures and images for: Optimization of artificial intelligence models for prediction of new-onset cardiovascular disease in patients with arterial hypertension
Source: PLOS Digit Health. 2026 May 21;5(5):e0001441. doi: 10.1371/journal.pdig.0001441 (PMC13193449; doi:10.1371/journal.pdig.0001441)

S1 Fig: Example of a time series of a selected variable.

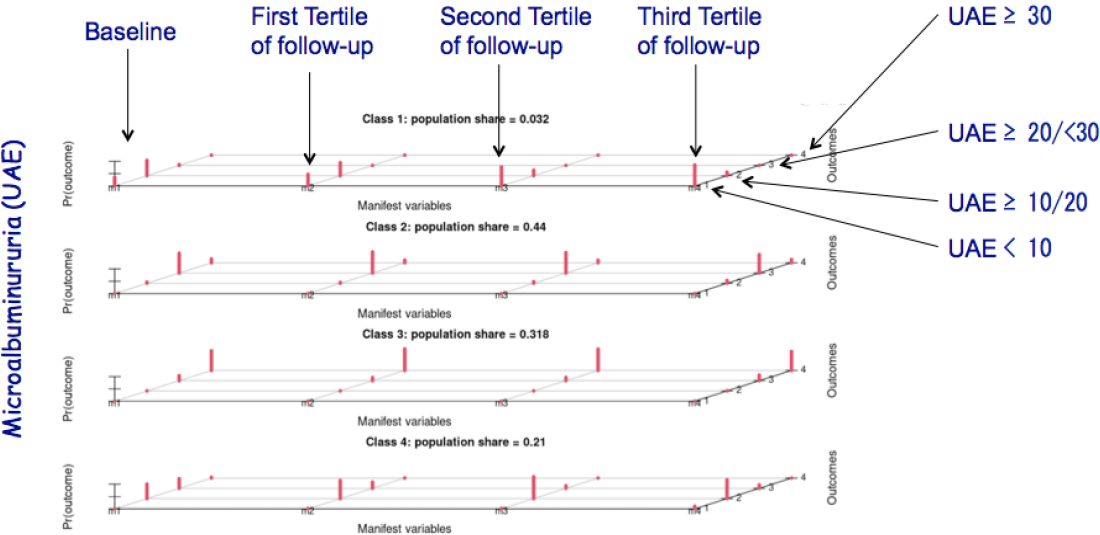

Supplement: S1 Fig — (PDF) [file pdig.0001441.s008.pdf]

S2 Fig: Calibration plot in internal validation.

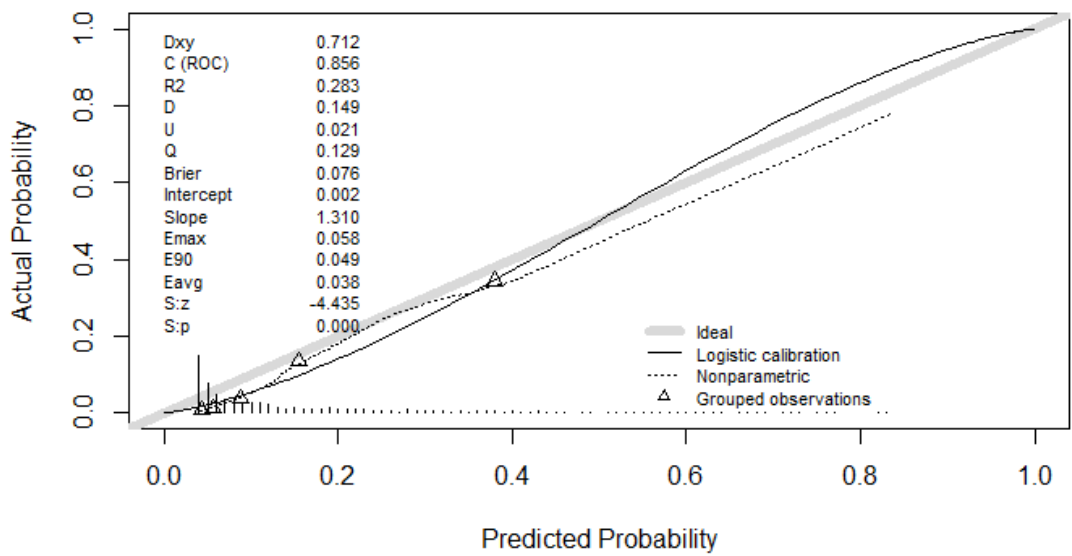

Supplement: S2 Fig — (PDF) [file pdig.0001441.s009.pdf]
