# Supplementary material for: Optimization of artificial intelligence models for prediction of new-onset cardiovascular disease in patients with arterial hypertension
Source: PLOS Digit Health. 2026 May 21;5(5):e0001441. doi: 10.1371/journal.pdig.0001441 (PMC13193449; doi:10.1371/journal.pdig.0001441)

S3 Fig: Decision curve analysis in internal validation. Net benefit of using the XGBoost model across threshold probabilities compared with "treat-all" and "treat-none" strategies; higher net benefit indicates greater clinical utility at a given threshold.

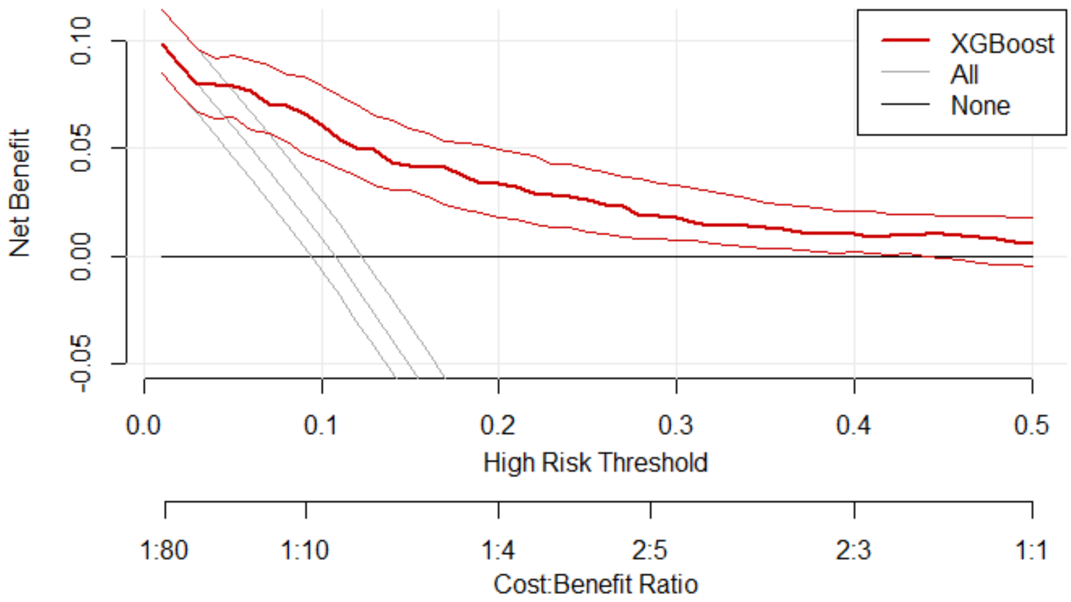

Supplement: S3 Fig — Net benefit of using the XGBoost model across threshold probabilities compared with “treat-all” and “treat-none” strategies; higher net benefit indicates greater clinical utility at a given threshold. (PDF) [file pdig.0001441.s010.pdf]
