# Supplementary material for: Optimization of artificial intelligence models for prediction of new-onset cardiovascular disease in patients with arterial hypertension
Source: PLOS Digit Health. 2026 May 21;5(5):e0001441. doi: 10.1371/journal.pdig.0001441 (PMC13193449; doi:10.1371/journal.pdig.0001441)

S4 Fig: Cumulative gain (lift) curve in internal validation. Cumulative proportion of observed CVD events captured as patients are ranked from highest to lowest XGBoost-predicted risk. The dashed diagonal indicates random selection; deviation above it reflects event enrichment in the highest-risk strata.

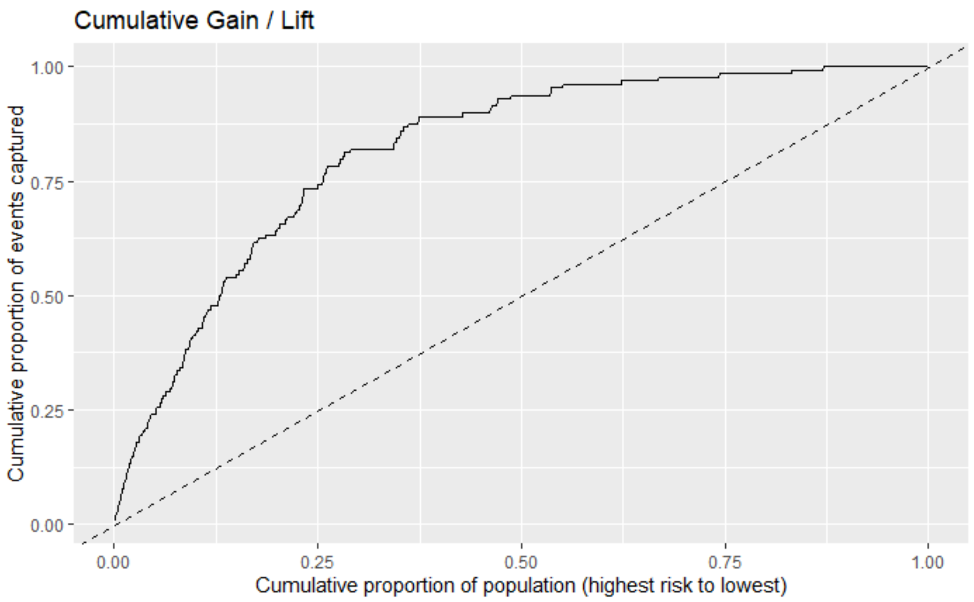

Supplement: S4 Fig — Cumulative proportion of observed CVD events captured as patients are ranked from highest to lowest XGBoost-predicted risk. The dashed diagonal indicates random selection; deviation above it reflects event enrichment in the highest-risk strata. (PDF) [file pdig.0001441.s011.pdf]
