# Supplementary material for: Optimization of artificial intelligence models for prediction of new-onset cardiovascular disease in patients with arterial hypertension
Source: PLOS Digit Health. 2026 May 21;5(5):e0001441. doi: 10.1371/journal.pdig.0001441 (PMC13193449; doi:10.1371/journal.pdig.0001441)

S5 Fig. Kaplan–Meier curves of event-free survival stratified by predicted risk categories (low vs. high risk) according to the XGBoost model. Risk groups were defined using the ROC-derived optimal cut-off. Differences between groups were assessed using the log-rank test (p < 0.0001).

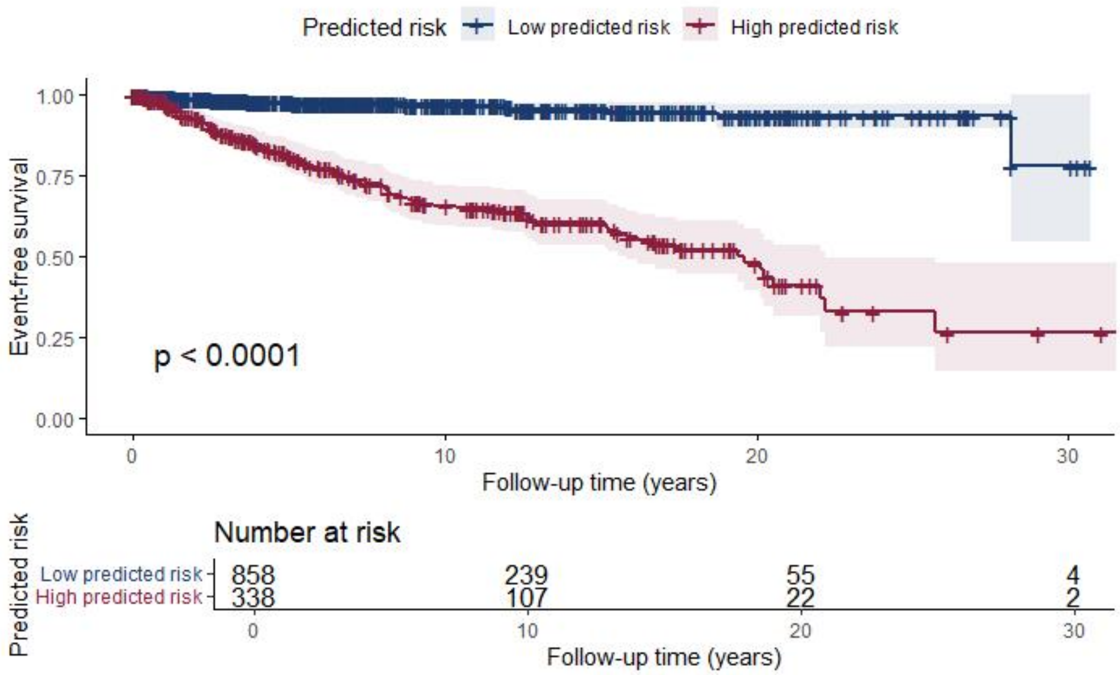

Supplement: S5 Fig — Risk groups were defined using the ROC-derived optimal cut-off. Differences between groups were assessed using the log-rank test (p = 0.0001). (PDF) [file pdig.0001441.s012.pdf]
